# Evaluation of a text-mining application for the rapid analysis of free-text wildlife necropsy reports

**DOI:** 10.1371/journal.pone.0337720

**Published:** 2025-11-25

**Authors:** Stefan Saverimuttu, Kate McInnes, Kristin Warren, Lian Yeap, Stuart Hunter, Brett Gartrell, An Pas, James Chatterton, Bethany Jackson

**Affiliations:** 1 New Zealand Center for Conservation Medicine, Auckland Zoo, Auckland, New Zealand; 2 Centre for Biosecurity and One Health, Harry Butler Institute, Murdoch University, Perth, Australia; 3 Department of Conservation/Te Papa Atawhai, Nelson, New Zealand; 4 Centre for Terrestrial Ecosystem Science and Sustainability, Harry Butler Institute, Murdoch University,; 5 Institute of Veterinary, Animal and Biomedical Sciences, Massey University, Wellington, New Zealand; Zydus Research Center, INDIA

## Abstract

The ability to efficiently derive insights from wildlife necropsy data is essential for advancing conservation and One Health objectives, yet close reading remains the mainstay of knowledge retrieval from ubiquitous free-text clinical data. This time-consuming process poses a barrier to the efficient utilisation of such valuable resources. This study evaluates part of a bespoke text-mining application, DEE (Describe, Explore, Examine), designed for extracting insights from free-text necropsy reports housed in Aotearoa New Zealand’s Wildbase Pathology Register. A pilot test involving nine veterinary professionals assessed DEE’s ability to quantify the occurrence of four clinicopathologic findings (external oiling, trauma, diphtheritic stomatitis, and starvation) across two species datasets by comparison to manual review. Performance metrics—recall, precision, and F1-score—were calculated and analysed alongside tester-driven misclassification patterns. Findings reveal that while DEE (and the principals underlying its function) offers time-efficient data retrieval, its performance is influenced by search term selection and the breadth of vocabulary which may describe a clinicopathologic finding. Those findings characterized by limited terminological variance, such as external oiling, yielded the highest performance scores and the most consistency across application testers. Mean F1-scores across all tested findings and application testers was 0.63–0.93. Results highlight the utility and limitations of term-based text-mining approaches and suggests that enhancements to automatically capture this terminological variance may be necessary for broader implementation. This pilot study highlights the potential of relatively simple, rule-based text-mining approaches to derive insights natural language wildlife data in the support of One Health goals.

## Introduction

Wildlife necropsy data sources are important repositories of information that can contribute to global health and conservation objectives [1–5]. These objectives include the intrinsic ecological and cultural value of species, which underscore animal and human health through functioning and resilient ecosystems. These in turn, provide for global health and security through ecosystem goods and services [6,7]. In 2022, the (belated) addition of the United Nations Environment Program (UNEP) to the tripartite of the World Organisation for Animal Health (WOAH, founded as OIE), the World Health Organisation (WHO), and the Food and Agriculture Organisation (FAO), resulted in the development of the quadripartite “One Health Joint Plan of Action” [8]. This was a global call to operationalise a more nuanced and balanced One Health approach, with improved recognition of the specific value of healthy environments and wildlife populations to the global One Health agenda [9]. Alongside the growing body of literature on ecological countermeasures for pandemic emergence [10], the global focus is on the benefits of prevention [11], followed by timely recognition and mitigation of health threats. Passive or convenience wildlife health data (such as in necropsy databases) are implicitly recognised in Actions 2.2 and 2.3 of the “One Health Joint Plan of Action”, which focus on surveillance systems to aid prevention and early response mechanisms in all health domains. However, this applied use of wildlife necropsy data to achieve early intervention goals has been hampered by the time and human-capital needed to access and review such data, as well as interpret it in light of inherent sampling and statistical biases [2].

Knowing what afflicts wildlife at the point of death can drive hypotheses and research into threats encountered at the population level [12], supporting the development of evidence-led mitigation strategies [4]. Yet, the methods by which wildlife necropsy data are gathered and stored at least in part create a barrier between data acquisition and its utilisation in real-time. The oft-shared environments of livestock, wildlife, and humans, create opportunities for pathogen flow [13–17], as brought to the fore of the public zeitgeist with the onset of the SARS-CoV-2 pandemic [12]. Despite the potential for wildlife necropsy data to detect and inform these events, it appears common that the insights from such data are not always obtained. Depending on the sophistication of data extraction and review processes, they may be relegated to the grey literature, held as local knowledge [18], or perhaps simply not derived.

Storage formats for wildlife necropsy data likely vary along a spectrum from heavily standardised forms dominated by picklists to being predominantly free-text [19] as in the database represented here. However, even in more standardised entry formats, free-text sections are likely required to capture the nuances of clinical assessments which may be lost in rigid coding or classification systems [20]. Further uptake and efficient utilisation of coding systems can be slow, error prone, or expensive [21]. The persistence of free-text throughout clinical data storage means that manual close reading remains a common method of data extraction, which is recognised in the human medical literature as a time consuming and error prone approach [22,23]. The potential utility of wildlife necropsy reports, combined with these free-text barriers to access, is a conflict observed in other fields with masses of narrative data, leading to the development of computational means of data extraction [22,24,25]. Overcoming these barriers is necessary for wildlife necropsy databases to realise their potential as early-warning systems for ecological change that may threaten species, ecosystems, or have cross-species epidemic or pandemic potential.

Rapidly expanding in use across human medicine [22], politics [24], and finance [25], text-mining describes the extraction of structured data from unstructured natural language text [26], not unlike wildlife necropsy reports. While also increasingly being applied in the veterinary sphere, much of the published literature on text-mining in veterinary medicine focuses on evaluating how such applications perform relative to traditional human review [27–29]. In these applications, the human review of a dataset is treated as ‘gold standard’ with the relative performance of the text-mining application described using the familiar metrics of sensitivity, specificity, and derivations thereof. Outside of the veterinary community similar metrics are applied, albeit with different terminology. Termed ‘recall’ and ‘precision’, these metrics are entirely analogous to sensitivity and positive predictive value and ubiquitous in the data science literature when evaluating the performance of a text-mining application. Additionally, calculation of an *F1-score* by combination of recall and precision is often used to succinctly convey the overall effectiveness of information retrieval [30].

‘DEE’ (‘Describe, Explore, Examine’) is a text-mining based, online application, built to facilitate the extraction of clinicopathologic data from free-text necropsy reports downloaded from the national wildlife pathology database of Aotearoa New Zealand, the Wildbase Pathology Register. A summary of the applications pertinent functions is given in the methods section of this paper. Source files for the application can be accessed via the GitHub repository at the following URL: https://github.com/SavStefan/TM-App, alongside an anonymised sample data file. While the application itself has other functions, here we use the metrics of recall, precision, and F1-score in a pilot test of one aspect of its capability. Specifically, the ability to retrieve cases of specified cliniopathologic findings from two collections of real-world necropsy reports, including variation in output based on user. In this context, ‘*clinicopathologic findings’* are defined as descriptive terms used in necropsy reports to denote observable pathological changes or diagnostic features identified during postmortem examination.

While DEE only functions with outputs of the Wildbase Pathology Register, the premise of the underlying code is relatively simple and common [22,24,26,31]. Through this preliminary evaluation of DEE, we review the suitability of these text-mining techniques for time efficient extraction of information from free-text wildlife necropsy data, with implications for broader use of such wildlife health databases across global health and conservation objectives.

## Methods

### Software

All data manipulation was performed in R through the R-studio interface alongside Microsoft Excel (Office 365).

### Necropsy data

Data for this trial was obtained from the Wildbase Pathology Register. A subset of necropsy records of kororā (blue penguin, *Eudyptula minor,* n = 361) and hoiho (yellow eyed penguin, *Megadyptes antipodes*, n = 911) were extracted from the database in comma separated value (.csv) format using its native search and download functionality (together referred to as the ‘test datasets’). The accessions in these datasets were individually, manually evaluated (by the lead author) to extract positive cases of clinicopathologic findings relevant to this pilot test and create a ‘reference dataset’ to which application tester results could be compared. Identification of these positive findings was based on adherence pre-determined descriptors for each finding as set out in Table 1.

**Table 1:** Clinicopathologic finding descriptions given for each dataset, and reference name of each finding in this study.

Test dataset	Description of clinicopathologic finding	Reference name of finding
Kororā	Oil contamination of the animal	‘Oil’
Kororā	Evidence of any traumatic injury	‘Trauma’
Hoiho	Diphtheritic stomatitis lesions observed	‘DipStom’
Hoiho	Evidence of clinically significant negative energy balance	‘Starve’

### The application

**Fig 1:**
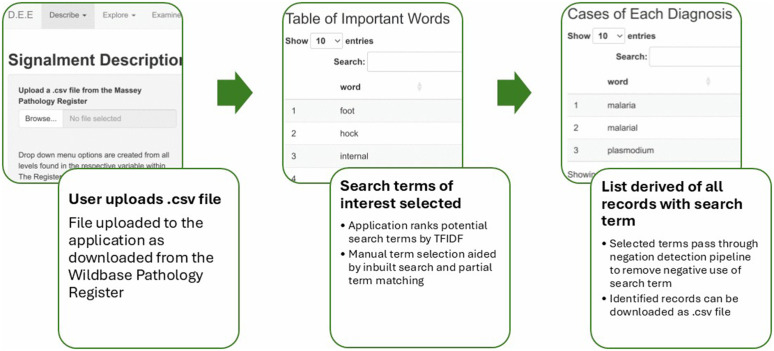
Summarised workflow of the ‘Examine’ section of DEE. DEE is an application designed for the rapid extraction of clinicopathologic data from accessions stored within the Wildbase Pathology Register of Aotearoa New Zealand. Selected illustrative screenshots and accompanying text outlines the workflow of the section of the application titled ‘Examine’ aimed at quantifying occurrences of clinicopathologic findings as specified by a user.

DEE allows the user to upload a.csv file containing aggregations of necropsy reports downloaded from the Wildbase Pathology Register. The application’s workflow is divided into three sections titled ‘Describe’, ‘Explore’, and ‘Examine’ (Fig 1). The ‘Describe’ section presents an interactive dashboard of signalment and submission characteristics of the uploaded dataset. ‘Explore’ contains two distinct methods of visually representing how common themes within the uploaded dataset may relate to each other, by analysing which words are used together within reports. These sections aim to provide a user with a quantitative overview of the population of the dataset, and a qualitative overview of common findings. The ‘Examine’ section aims to rapidly quantify the occurrence of user selected clinicopathologic findings from all uploaded necropsy records. It is this ‘Examine’ function of the application that is evaluated within this work.

Within the ‘Examine’ section, a user selects search terms of interest from a list of potential terms identified from records uploaded to the application. By default, the application derives the search term list by collating the top 40 words from each necropsy record with the highest term frequency to inverse document frequency ratio (TFIDF). As the name implies, this is a ratio of how frequently each word is found in an individual record to how frequently it is seen in all the uploaded records. Widely used in text-mining, TFIDF provides a statistical estimation of which words in each necropsy report likely contribute to the overall meaning contained within the report [32]. Users can scroll through this search term list or use the in-built partial word matching functionality to find and select search terms of interest. When a term of interest is selected the application then searches all the uploaded records for use of that term without a negator word (e.g. not, never, no etc) in the same phrase, a form of ‘rule-based negation detection’ [33,34]. A ‘phrase’ is defined in the application logic as the first use of punctuation before and after the selected search term within a ten-word window. The total number of unique records identified from all selected search terms is then displayed in a textbox. These records can then be downloaded in.csv format.

### Selection of application testers

Application testers were recruited using a purposive sampling approach, through the authors professional networks. Potential testers were invited via email with a brief study overview and estimation of time commitment. All potential testers were either veterinarians or veterinary pathology professionals actively working in the field of zoo and/or wildlife medicine who would, through the course of their regular work, have experience interpreting and/or writing veterinary necropsy reports. No geographic restrictions were used when soliciting testers. All respondents who expressed interest in participation were then invited to individual or small group (3 people maximum) meetings where they would be introduced to the application and complete the testing procedure. In most cases this meeting was held online utilising commercial video conferencing platforms. A single ‘in-person’ meeting was held with two testers. In total nine testers, working across 4 countries (Australia, New Zealand, United Kingdom, and Cambodia), completed the application testing protocol.

### Application testing protocol

Each testing meeting began with a ten-minute introductory presentation on the motivations behind the development of this app, its broad functionality, and the aims of the testing procedure. This was followed by an approximately 15 minute in-browser walkthrough of the application. This walkthrough utilised a demonstration dataset also obtained from the Wildbase Pathology Register but with no common accessions to the two test datasets.

Testers were then supplied with the hoiho and kororā test datasets as well as a separate ‘test recording’ word document via email (see S1 File). The ‘test recording’ document featured a table which specified the two clinicopathologic descriptions for each test dataset alongside a pre-filled example, as well as brief written instructions of the testing protocol.

The testers were instructed to use the application’s ‘Examine’ page to download all records relating to each clinicopathologic descriptor from their respective dataset. For each clinicopathologic descriptor, the testers were also instructed to list all the search terms they selected in the record identification process as well as the total number of records identified. These data were recorded in the ‘test recording’ document. Testers then submitted the completed test recording document as well as the four.csv files downloaded from the application containing all records identified for each clinicopathologic finding through their use of the application. The descriptors chosen for this pilot were selected intending to explore how the application handles terms that vary in both frequency and specificity. Descriptors, like “oil,” are narrowly defined and unlikely to be expressed in alternative ways, while others, such as “starvation,” may be represented by a range of synonymous terms (e.g., emaciated, low body condition, skinny).

As the application testing process was conducted immediately after the demonstration, within the scheduled meeting, the lead author remained readily available for testers to seek technical clarification on the application’s functions as they carried out the testing protocol.

### Data analysis

#### Initial processing.

All data from the test recording documents were transposed into commercial spreadsheet software (‘Google sheets’) (complete datafile available in.csv format in S2 File). The total number of search terms, as well as the number of three letter word stems (i.e. any sequence of three letters which may serve as the start of one or multiple words) encompassing these search terms was also calculated and recorded for each case. A simple R-Shiny application was then employed to compare the accession numbers of records in the tester’s datasets to those of the relevant reference dataset and output the following:

1. Number of records related to the clinicopathologic finding in the reference dataset, that were retrieved by the tester (True Positives)
2. Number of records unrelated to the clinicopathologic finding in the reference dataset, that were retrieved by the tester (False Positives)
3. Number of records related to the clinicopathologic finding in the reference dataset, that were not retrieved by the tester (False Negatives)

The complete code for this processing tool can be found in the file titled ‘Analysis app.R’ at the following GitHub repository: https://github.com/SavStefan/Evaluation-of-a-text-mining-application.

Datasets of the False Positives and False Negatives for each comparison were downloaded to enable misclassification analysis.

#### Misclassification analysis.

Misclassifications were categorised according to Table 2 below, with each individual record annotated with the letter code most closely describing the reason for misclassification. To expedite the classification coding process, the largest false positive and false negative datasets for each clinicopathologic finding were manually reviewed to determine the cause of misclassifications for each tester. An R-script was then used to automatically annotate all other tester false positive and false negative datasets for each clinicopathologic finding, by comparison to these first datasets. Any individual accessions which were not automatically annotated by this process (as they were not present in the manually annotated dataset) were then read and manually annotated. The complete code for this processing tool can be found in the file titled ‘Annotating app.R’ at the following GitHub repository: https://github.com/SavStefan/Evaluation-of-a-text-mining-application.

**Table 2:** Table of misclassification coding structure. Codes in the left-hand column were given to individual records misclassified by the application according to the false positive and false negative code definitions and examples described.

Code	Description	False negative code definition	False Negative example	False positive code definition	False positive example
R	Record structure	Record structure lacks space for the provision of necessary details.	None found	Record structure led to the inclusion of details creating a false positive.	History notes specimen found during oil spill response but not oiled in necropsy findings. Results in false positive with search term ‘oil’
S	Search term use	Search term encompassing multiple true positive reports not used.	Using only emaciation and closely related words results in false negatives where clinically significant negative energy balance is described as ‘starvation’.	Selection of a search term which does not describe the clinicopathologic finding of interest.	‘Reserve’ selected as search term however used to describe both positive and negative cases of negative energy balance.
I	Interpretation	Clinicopathologic finding was included in manual review by interpretation of the record rather than specific reference to a clinicopathologic finding.	Animal listed as of exceptionally low body condition so interpreted as emaciated however not specifically mentioned in necropsy report as ‘emaciated’ (or synonyms).	Clinicopathologic finding was excluded from manual review by interpretation of the record despite specific reference to a clinicopathologic finding.	Evidence of traumatic injury described in report but suggested to be due to post-mortem events so excluded in manual review but included by the application.
G	Grammatical error	Lack of punctuation includes negation word in the negation window while not strictly in the same sentence.	‘Stomatitis not found but multiple lacerations present on the body’ – a lack of appropriate punctuation (in this case a comma following ‘found’) applies the negator to the positive finding.	Inadvertent punctuation separates search term from its negator.	‘No, signs of oiling’ – Inadvertent comma separates finding from negator.
T	Typography	Typographical variant or error not selected as a search term.	Common misspellings of ‘diphtheritic’ creates a multitude of typography, all of which would need to be selected to encompass all positive cases. Any missed will be false negatives.	Typographical variant or error causes finding to escape negation detection.	‘no signs of trauma/ predation’ – app interprets ‘/’ as sentence end so does not apply negator to ‘predation’.
U	Unknown	Cause of false negative could not be determined.		Cause of false positive could not be determined.	

### Performance metrics

The following three performance metrics were calculated for data retrieval of all clinicopathologic findings, for each tester, according to the formulae provided (as has been described in similar applications see [23,35,36]):

1. Recall (synonymous with ‘sensitivity’):

$$\frac{True\;Positive}{True\;Positive+False\;Negative}$$

2. Precision (synonymous with ‘positive predictive value’):

$$\frac{True\;Positive}{True\;Positive+False\;Positive}$$

3. F1-score (the harmonic mean of Recall and Precision):

$$2\times \frac{Precision\times Recall}{Precision+Recall}$$

### Search term count and word stem relationships

The association of search term counts (the total number of search terms used) and word stems with performance metrics was evaluated with descriptive statistics.

### TFIDF analysis

Each tester was instructed to perform the application test under its default settings. Specifically, this means that selectable search terms are generated by collating the top 40 highest TFIDF words from each record uploaded to the application. To explore the effect of reducing this value on the applications performance, each tester’s tests were repeated (by the author), using their recorded search terms with TFIDF values of 20, 10 and 5. Results from these repeated analyses were then recorded in the same spreadsheet and subject to the same processing as the raw data (as described above). The misclassification analysis was not performed on these repeat datasets.

## Results

All nine testers completed the application testing protocol in 20–40 minutes.

### Misclassification analysis.

Overall, the search terms selected by testers was the reason for most misclassifications, leading to 67.6% of the false positive records, and 44.7% of the false negative records (Table 3). Within false positive misclassifications, interpretation was the second highest cause of these records being retrieved erroneously, whereas the reason for 24.9% of false negative records was unknown (unable to be determined). Record structure, and typographical or grammatical causes of misclassification were either absent or minimally recorded.

**Table 3:** Quantification of misclassification categorisation. Misclassification categories quantified across all testers and clinicopathologic findings for false positives and false negatives in a pilot test of the text-mining application; DEE, designed for the rapid retrieval of necropsy data from free-text reports within the Wildbase Pathology Register of Aotearoa New Zealand.

Misclassification code	False negative % (x/n)	False positive % (x/n)
**R** *Record structure*	0	4.0 (53/1341)
**S** *Search term use*	67.6 (812/1201)	44.7 (599/1341)
**U** *Unknown*	24.9 (299/1201)	10.0 (134/1341)
**I** *Record Interpretation*	3.9 (47/1201)	40.6 (545/1341)
**G** *Grammatical error*	0	0
**T** *Typography*	3.6 (43/1201)	0.7 (10/1341)

**Fig 2:**
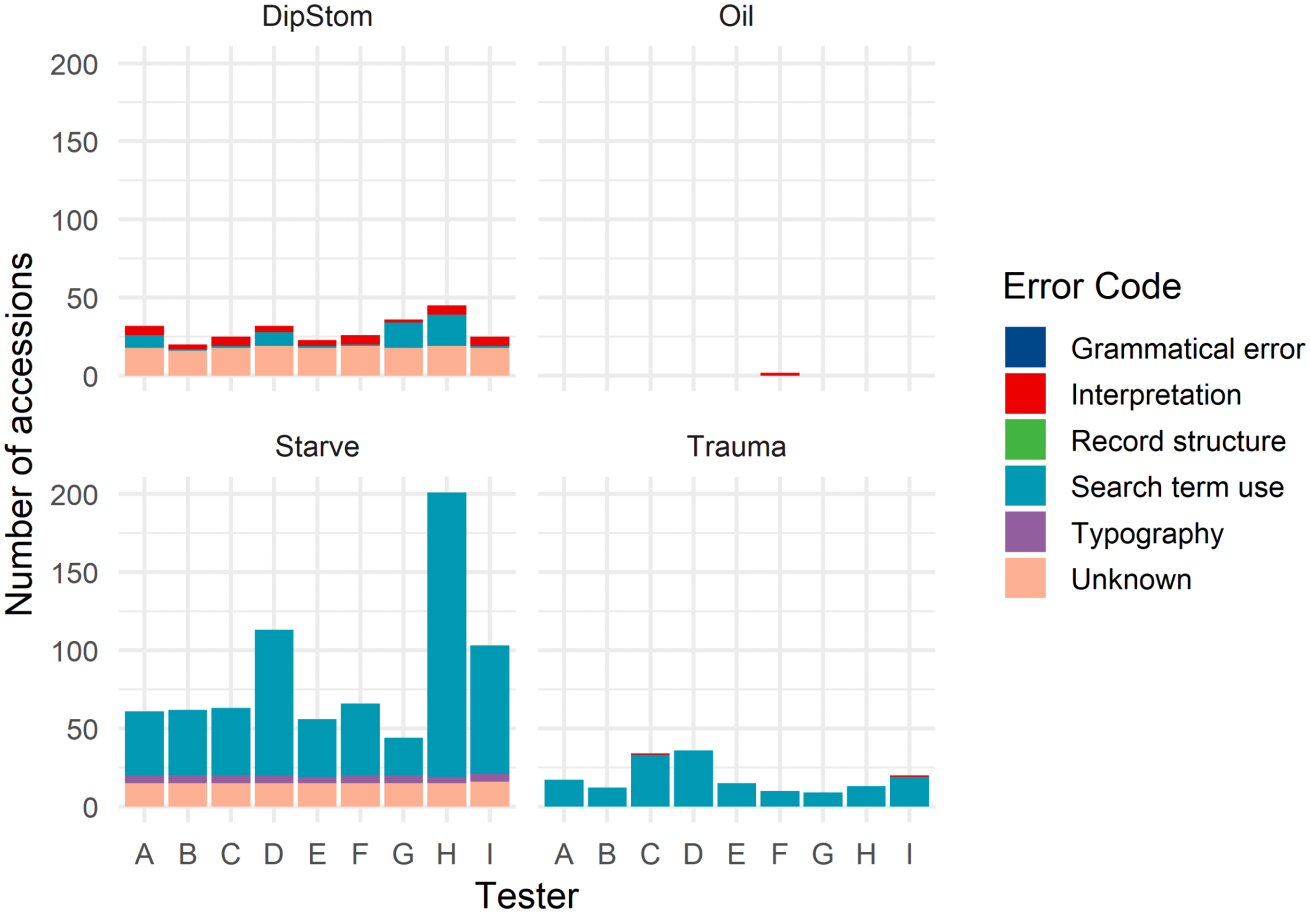
Bar charts of false negative misclassifications for tested clinicopathologic findings across all testers. Number of false negative accessions extracted (y-axis) by each anonymised tester (x-axis) stacked by reason for misclassification (key), for each tested clinicopathologic finding (as labelled) during pilot testing of the application, DEE, for extraction of necropsy data from the Wildbase Pathology Register.

**Fig 3:**
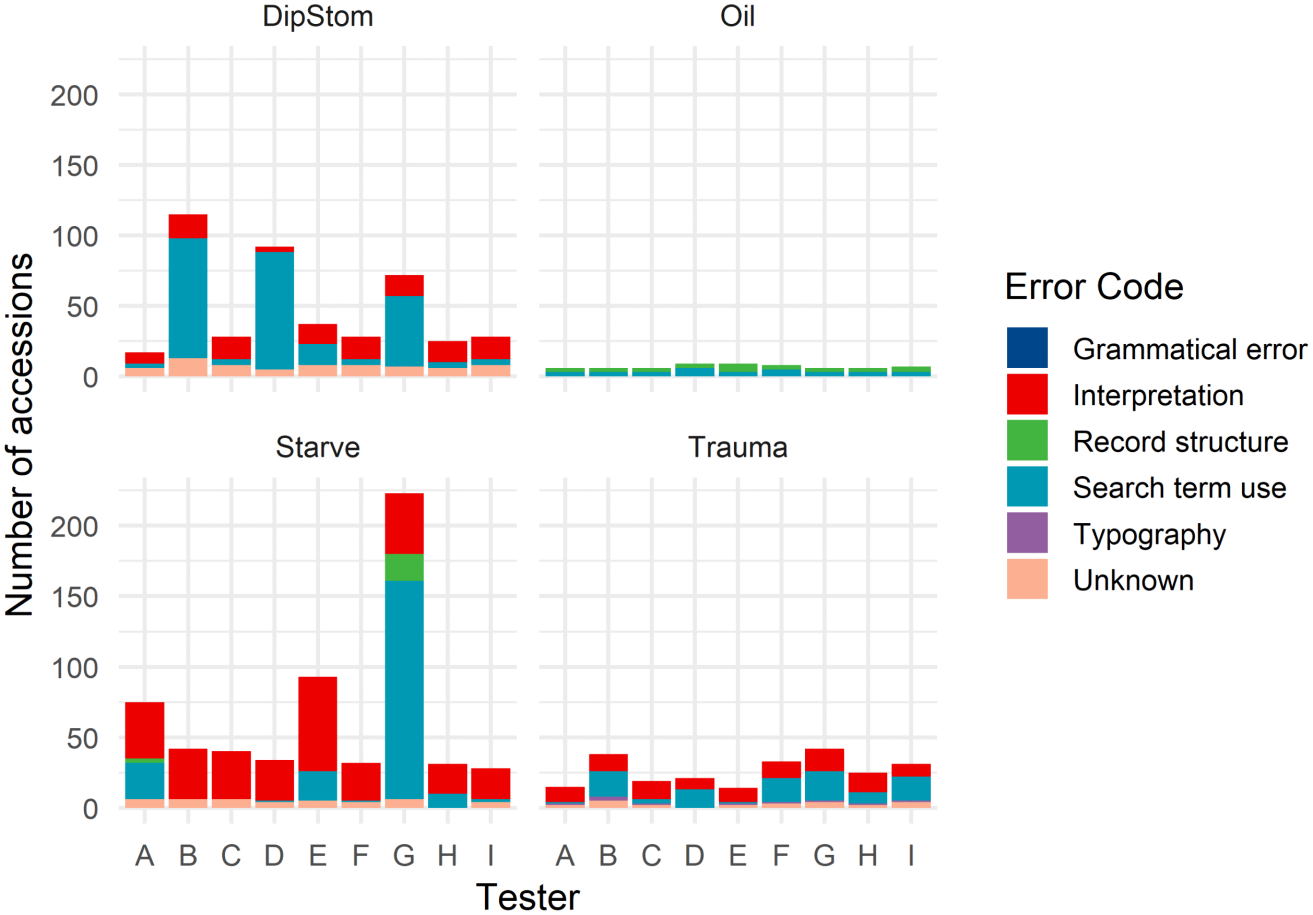
Bar charts of false positive misclassifications for tested clinicopathologic findings across all testers. Number of false positive accessions extracted (y-axis) by each anonymised tester (x-axis) stacked by reason for misclassification (key), for each tested clinicopathologic finding (as labelled) during pilot testing of the application, DEE, for extraction of necropsy data from the Wildbase Pathology Register.

Causes of false positive and false negative records varied between testers for any one clinicopathologic finding, and within testers across the tested clinicopathologic findings. The distributions of these misclassifications and their assigned categories are described in Fig 2 and Fig 3 below.

### False negative records

Broadly, false negative (missed) records were most common in the ‘Starve’ finding (64%, 769/1201), and least common in ‘Oil’ (0.2%, 2/1201). For the ‘Starve’ and ‘Trauma’ findings, the search terms used drove the majority of the false negative misclassifications (‘Starve’: 76.7%, 590/769 records, ‘Trauma’: 98.8%, 164/166 records). Within the ‘DipStom’ finding, the majority of false negative misclassifications were in the ‘unknown’ category (61.7%, 163/264), followed by search term used (22.0%, 58/264). Within the ‘Starve’ finding, tester “H” returned the highest proportion of all false negative records (25.6%, 197/769), primarily because of records that were missed due to inappropriate search term selection (92.4%, 182/197).

### False positive records

Causes of false positive (erroneously included) records were more variable across testers and clinicopathologic findings. Record structure and search term use accounted for all false positive misclassifications within the ‘Oil’ finding, whereas record interpretation accounted for 40.6% (545/1341) of false positive misclassifications in all other clinicopathologic findings. Inappropriate search term usage contributed to false positive misclassification variably across testers, with tester “G” deriving the majority (71.8%, 155/216) in the ‘Starve’ finding, compared to three testers sharing the majority in the ‘DipStom’ finding (86.5%, 218/252).

### Performance metrics

**Fig 4:**
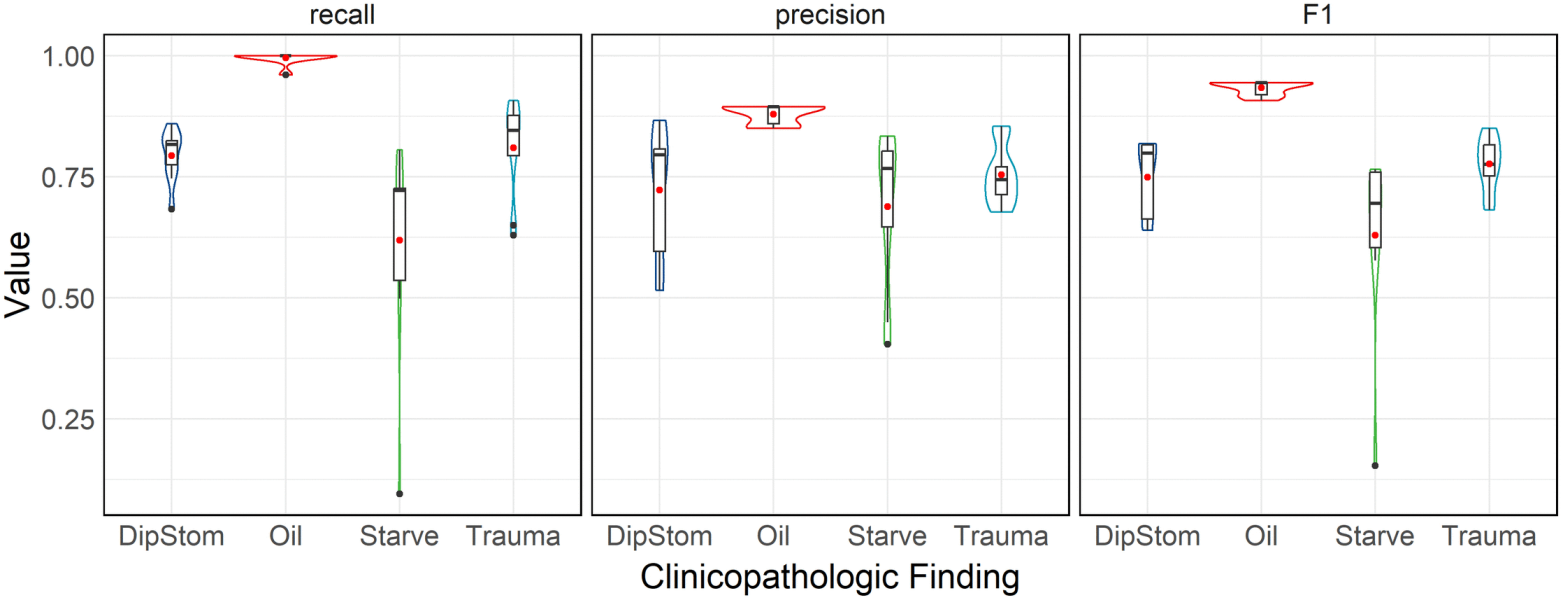
Violin plots of performance metrics across all testers. Recall, precision, and F1-score (as labelled) distributions (y-axis), for each tested clinicopathologic finding (x-axis), across all testers of the application for extraction of necropsy data from the Wildbase Pathology Register, DEE.

Violin plot and tabulated values of statistical metrics of application performance (recall, precision, and F1-score) across testers are presented in Fig 4 and Table 4 respectively.

**Table 4:** Metrics of application performance. Mean, standard deviation, and range of recall, precision, and calculated F1-score of all four tested clinicopathologic findings across all testers of the application for extraction of necropsy data from the Wildbase Pathology Register, DEE.

	*Recall*			*Precision*			*F1-Score*		
	*Mean*	*SD*	*Range*	*Mean*	*SD*	*Range*	*Mean*	*SD*	*Range*
*DipStom*	0.79	0.05	0.68-0.86	0.72	0.13	0.51-0.87	0.75	0.08	0.64-0.82
*Oil*	1	0.01	0.96-1	0.88	0.02	0.85-0.89	0.93	0.01	0.91-0.94
*Starve*	0.62	0.22	0.09-0.81	0.69	0.16	0.40-0.83	0.63	0.19	0.15-0.77
*Trauma*	0.81	0.10	0.63-0.91	0.75	0.06	0.68-0.85	0.78	0.06	0.68-0.85

Across all testers, the ‘Oil’ finding had the highest mean and lowest standard deviation for the metrics of recall, precision, and F1-score. Greater variability between testers was seen for the other findings, with the ‘Starve’ finding having both the lowest mean F1-score and largest standard deviation.

### Search term analysis

Findings of ‘Oil’ and ‘DipStom’ had the lowest number of word stems and equivalent word stem range despite ‘DipStom’ having more than double the mean number of terms used (Table 5).

**Table 5:** Statistics of search terms and stems. Mean, standard deviation, and range of the number of selected search terms and number of 3 letter word stems derived from these search terms, across testers and clinicopathologic findings in the pilot test of an application for extraction of necropsy data from the Wildbase Pathology Register, DEE.

	Terms			Stems		
	*Mean*	*SD*	*Range*	*Mean*	*SD*	*Range*
**DipStom**	11.5	3.5	6-18	2.3	0.9	1-4
**Oil**	5.1	2.1	1-9	1	1	1-4
**Starve**	9	2.1	6-12	4.1	1.8	2-8
**Trauma**	13.1	6.4	6-24	5.3	1.8	3-8

### TFIDF analysis

**Fig 5:**
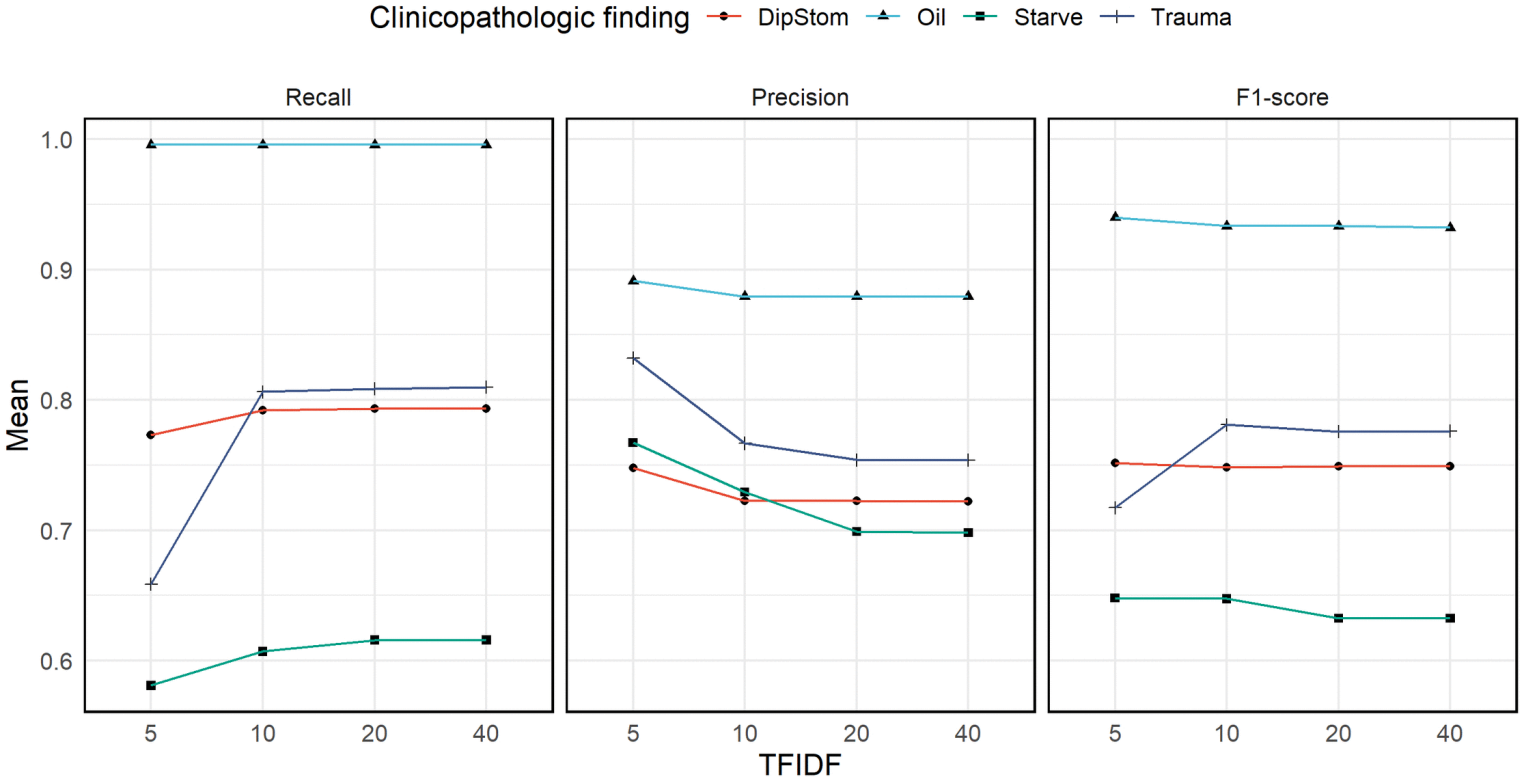
Line plots of performance metrics across TFIDF conditions. Mean (y-axis) of recall, precision, and F1-score (as labelled) of all testers, for each clinicopathologic finding (key), across the four TFIDF conditions (x-axis) tested in a pilot test of the application, DEE, designed for extraction of necropsy data from the Wildbase Pathology Register.

Across all findings and testers, increasing TFIDF appeared to increase mean recall and decrease mean precision, except for the ‘Oil’ finding which was largely unaffected by changes to TFIDF. The magnitude of this change was greatest comparing TFIDF 5 to TFIDF 10. The effect of these changes to precision and recall on F1-score was more variable. Mean F1-score of ‘Trauma’ findings increased substantially comparing TFIDF 5 to 10, while all others mean F1-score changes ranged from slight decreases to nothing with rising TFIDF (Fig 5).

The distribution of performance metrics across all testers, clinicopathologic findings, and each TFIDF condition can be found in S1 Fig.

## Discussion

Using a focus group pilot approach, we provide preliminary data on the performance of the bespoke text-mining application ‘DEE’, for the identification of selected clinicopathologic findings in wildlife necropsy records. While designed for the Wildbase Pathology Register of Aotearoa New Zealand, the application’s functions are a case study for time efficient extraction of information from free-text wildlife health data more broadly. By streamlining data processing, this capability enables more responsive surveillance, allowing for timely detection of alterations in the frequency or type of clinicopathologic findings being observed within a population. In turn, the detection of these alterations may may act as an early warning system for ecological change and/or disease emergence.

Overall, we posit that the application has potential for resource efficient data extraction, taking 20–40 minutes per tester to derive a list of records aligned to two clinicopathologic findings each of two species datasets. However, variability across testers was observed in the performance metrics calculated (recall, precision, and F1-score). Search terms selected by the tester had the greatest influence on either failure to retrieve (false negative), or inappropriate retrieval of (false positive) records, for a given clinicopathologic finding. Searching for clinicopathologic findings that have limited word search options (the ‘Oil’ finding for example) performed the best across recall, precision, and F1-scores, independent of tester. Collectively these results provide insight into factors that may optimise the output of such a tool, as well as some inherent limitations of the principles applied that should be considered for the development of similar tools in other contexts.

A primary objective of wildlife necropsy databases is to document the occurrence and frequency of specific clinicopathologic findings, which can facilitate hypothesis generation or analysis of common epidemiological risk factors such as species, age classification, timing, and location. By inference, a primary outcome of DEE is the tester’s ability to correctly identify all records that fit with a specific clinicopathologic search query, serving the broader surveillance objectives of such passive datasets, that include early detection and qualification of unusual health events in wildlife [37]. Given this, misclassification of records is a major risk of any automated record retrieval system, owing to the potential to erroneously reject records (false negative) or include records (false positive). In this pilot study, search term selection by the tester was the largest contributor to both false negative (44.7%) and false positive (67.5%) record retrieval. Within the false negative records, these misclassifications were almost entirely within the findings of ‘Starve’ and ‘Trauma’, which have numerous synonymous terms and word stems, with none in the ‘Oil’ finding. Consistent with text-mining of human electronic medical records [23], when a low variety of words are used to report a given condition, there is a higher chance that all relevant search terms are selected by a tester to derive the dataset of interest. Further, we saw that short phrases may replace single word descriptors of a specific finding, such as describing a ‘starving’ animal as of ‘low body condition’. This suggests that expanding the search term selection from single words to multiple word ‘n-grams’ [31] may improve the performance of these text-mining techniques in some scenarios. In contrast, false positive misclassifications due to search term selection were seen across all clinicopathologic findings, though there was substantial inter-tester variability within each tested finding. We hypothesise that having a greater contextual understanding of a dataset would minimise the impact of search term-based misclassifications by informing more appropriate search term discrimination. The varied geographic background of testers meant that most had limited familiarity with the context of the species chosen in Aotearoa New Zealand. In contrast to this geographic diversity, the purposive sampling approach used to invite application testers meant that each was a veterinary professional, presenting a significant limitation to these results. By its nature, wildlife health sits at the interface of medicine and ecology [38]. The inclusion of professionals in the vastly broader range of disciplines within wildlife health, or perhaps even interdisciplinary collaboration, may serve to fill the gap of contextual knowledge required to optimise results from such a text-mining application. Importantly, our results suggest that if the purpose of a data search is to detect specific exotic or important wildlife diseases, optimising recall to minimise false negative records is key, which is related to the linguistic variability of the clinicopathologic finding in question, likely alongside a users’ familiarity with the dataset.

Misclassifications attributed to record interpretation were abundant within the false positives (40.8%) while only having a small contribution to the false negatives (3.9%). The false positives here refer to records that were manually reviewed and excluded because the findings were considered clinically insignificant. This is reflective of the subjective nature of any attempt to rigidly categorise free-text medical data, as needs to be done during a manual necropsy review. Here, there is a parallel to diagnostic coding systems seen in human medicine where a loss of granularity from the free-text reports and subjectivity in the assignment of codes is a recognised limitation [39,40]. We suggest that the use of text-mining techniques allows this discrimination to take place at the point of data extraction, rather than data input as with medical coding systems. Records which are not relevant to a particular question (i.e. false positives) can then be manually filtered from this, more manageable dataset. Ultimately, this results in extraction of a dataset that is more closely aligned with the objectives of a particular analysis rather than being beholden to the judgments of the original report author as is the case when collating pre-coded records.

Other causes of misclassification represented relatively small contributions to all false positive and false negatives. Of note, misclassifications due to record structure only generated false positives within the ‘Oil’ finding. This was because of the frequent mention of an important event (a commercial oil spill) within the history section of records where the event had no impact on the pathology observed. Further consideration of the record structure in the design of a text-mining application may mitigate this issue. Surprisingly, no misclassifications due to grammatical errors were found, highlighting the strength of the relatively simple rule-based negation detection employed in this application. While consistent with some previous research [34], limitations of this approach are recognised [33,34] and worth considering if applied more broadly. Finally, misclassifications whose cause could not be determined accounted for 24.9% (299/1201) of all false negatives and 10.0% (134/1341) of false positives, representing a substantial impact on the applications performance. These records showed some consistency across testers, suggesting a shared interaction between these reports and the application’s mechanics, however detailed inquiry into these reports was beyond the scope of this trial. As a whole, the diversity of misclassification causes found in this trial highlights the need to be vigilant for unforeseen emergent properties when combining text-mining techniques and free-text medical reports.

Similar to other studies comparing text-mining applications to results obtained by human review [30,41–43], variability in the calculated performance metrics was seen in the study presented here. The upper limits of the range of F1-scores (0.77–0.94) demonstrate that this tool, and by extension the underlying principles, has the potential to be a rigorous tool for data extraction dependant on the goals being pursued. The relatively low recall seen for the findings of ‘Starve’ and ‘Trauma’ demonstrated that such an application is less suited as the sole method to determine the presence or absence of linguistically complex clinicopathologic findings. For rare and linguistically complex findings, such as those associated with emerging syndromes, tools like this may offer a useful first-pass screening of necropsy datasets. Nonetheless, given the limitations identified in this pilot, absence of detection should not be interpreted as true absence without additional review of the dataset.. By contrast, the relatively high F1-score across testers of the ‘Oil’ finding demonstrates that when a clinicopathologic finding of interest is described by a constrained vocabulary, a tool such as this may be particularly useful for monitoring changes in incidence over time. Further, the total range observed across the calculated metrics is evidence that user context can have substantial impact on application performance. Despite some testers achieving rather underwhelming F1-scores for ‘Starve’ and ‘Trauma’, others achieved values more comparable to that seen with the ‘Oil’ finding. From this, we hypothesise that a user who has some contextual familiarity with the dataset being interrogated is likely to be able to use such a tool more reliably even when linguistically complex findings are the target. Further, total search term counts, or word stem counts seen across testers appeared to have no bearing on the calculated performance metrics. However, there does appear to be a relationship between the number of word stems which may describe a finding and the performance metrics. Across testers, the findings of ‘Oil’ and ‘DipStom’ had the lowest mean number of word stems used while having the highest mean F1-scores. Though our sample size of testers and clinicopathologic findings are small, this further supports that the text-mining approaches presented here are more suited to clinical findings described by a constrained vocabulary.

Finally, we reviewed the value of integrating TFIDF into a text-mining workflow for the extraction of information from the tested datasets. This is a statistical estimation of how important a word is to the meaning of a document within a collection. TFIDF has been widely applied in other knowledge extraction endeavours including document classification [44] and associative knowledge graphing [45], all leveraging the assumption that documents which share high TFIDF value words are likely related in meaning. We hypothesised that filtering selectable search terms to only those with a greater TFIDF value would result in improved precision, potentially at the expense of recall. While this appears to be true when comparing the mean TFIDF-5 and TFIDF-10 results, further increases in TFIDF yielded at most, modest increases in recall, with little to no effect on precision and F1-score. While only four clinicopathologic findings were tested here, this result suggests that the TFIDF measure of word importance may not be suitable for this use except when precision is absolutely prioritised so TFIDF may be severely restricted. As all the performance metrics showed only minor change between TFIDF-20 and TFIDF-40 we further suggest that alternative techniques are employed if intending to give a user finer control over the precision and recall of such an application.

Overall, this pilot study revealed a key limitation of the applications mechanics: variability in user selected search terms. It is this variability that drives the discrepancies in application performance metrics seen across the testers and clinicopathologic findings. This limitation likely stems from the structure of English medical language itself, resulting in multiple ways to describe the same phenomena [22,46–48]. This constraint may be overcome by broadening the search focus from specific words describing a finding to the meaning of words or even whole sentences which describe a finding [49,50]. Through statistical or machine learning means, the relationship in meaning between different words can be determined by analysing how they are used within sentences. While machine learning methodologies are commonplace for these tasks, they rely on pre-training with large datasets or existing ontologies to output accurate results [51]. Further these approaches may create tools that perform the best when applied to data most-similar to that on which it was based [39,52]. While specifically training a bespoke model for the purposes of mining free-text wildlife health reports is a possibility, utilising a statistical approach to calculate word or sentence semantics is likely to be far less resource-intensive and potentially more universally applicable depending on what source data is used to train a bespoke machine learning model [53].

## Conclusions and future directions

Pathway 3 of the global One Health Joint Plan of Action 2022–2026 focuses on leveraging technology to strengthen information systems and foster both the creation and sharing of knowledge [8]. The development of novel methods to overcome the resource barriers between data acquisition, data analysis, and decision making are key to addressing this Pathway. While the application and datasets reviewed here are specific to the Wildbase Pathology Register of Aotearoa New Zealand, this comparison of manual versus automated data extraction is a case study for the suitability of the underlying principles to be used in interrogation of similar free-text wildlife health data sources. More importantly, limitations identified in this study, including the need for diligent search term selection if recall is prioritised (e.g., in a surveillance setting), clearly indicate areas of future inquiry. A shift away from relatively simplistic term-based text-mining to semantic methods of analysis, be it statistical or machine learning, is recommended to overcome the limitations identified here. Further development in this area likely requires more aggressive financial input to organisations that collect such data, to prioritise issues of data management and knowledge sharing. This will enable time-sensitive cross-industry and stakeholder collaboration, so that such meticulously collected and stored data can be utilised to its fullest potential.

## Supporting information

S1 File Test recording document. Document sent to all testers of a bespoke text-mining application for the rapid extraction for clinicopathologic data from outputs from the Wildbase Pathology Register of Aotearoa New Zealand.
(DOCX)

S2 File All tester data. Compilation of results from each tester of a bespoke text-mining application for the rapid extraction for clinicopathologic data from outputs from the Wildbase Pathology Register of Aotearoa New Zealand.
(CSV)

S1 Fig Violin plot of all application test performance metrics. The distribution of performance metrics (right side labels and y-axis) across all testers (violin plots), clinicopathologic findings (x-axis), and each TFIDF condition (top labels) in a pilot test of the application DEE, designed for rapid extraction of necropsy data from the Wildbase Pathology Register of Aotearoa New Zealand.
(TIF)
