# Supplementary material for: Evaluation of a text-mining application for the rapid analysis of free-text wildlife necropsy reports
Source: PLoS One. 2025 Nov 25;20(11):e0337720. doi: 10.1371/journal.pone.0337720 (PMC12646400; doi:10.1371/journal.pone.0337720)
Supplement: S1 File — Document sent to all testers of a bespoke text-mining application for the rapid extraction for clinicopathologic data from outputs from the Wildbase Pathology Register of Aotearoa New Zealand. (DOCX) [file pone.0337720.s001.docx]

**DEE testing form**

*Instructions*

1. Go to <https://stefansav.shinyapps.io/necropsy-text-mining-2/>
2. Upload the dataset appropriate to the species of interest as denoted in the ‘Species’ column of the below table.
3. Click through to the ‘Examine’ section.
   1. Feel free to interact with the ‘Describe’ and ‘Explore’ sections along the way but you do not need them for the purposes of this exercise.
4. For each clinicopathologic description in the ‘Description’ column of the below table, utilise the word search feature to select relevant terms in the ‘Table of Important words’ of the application.
5. Record each selected word in the ‘Terms’ column of the below table
6. Once you have selected as many words as you like please download all the identified records using the ‘Download selected records’ button in the application.
7. Name this .csv file as such: *species_findingname_testerinitials.csv*. For example: *hoiho_stomatitis_ss.csv*.
8. Repeat this process for all four rows of the below table.
9. Please email this collection of four .csv files and this word document to: [ssav2852@gmail.com](mailto:ssav2852@gmail.com).
10. Feel free to include any additional feedback you would like in that email or within this document.
11. Once you have completed this test and emailed me your generated files my collaborators and I ask that you kindly delete the .csv files.

| Species | Description | Terms | Number of cases |
| --- | --- | --- | --- |
| Tawaki | Specimen quality compromised at the time of necropsy | Decomposition, decompose, decomposed, decomposing, freeze-thaw, freezing, freezer | 16 |
| Korora | Oil contamination of the animal |  |  |
| Korora | Evidence of any traumatic injury |  |  |
| Hoiho | Diphtheritic stomatitis lesions observed |  |  |
| Hoiho | Evidence of clinically significant negative energy balance |  |  |
