# Supplementary figures and images for: Evaluation of a text-mining application for the rapid analysis of free-text wildlife necropsy reports
Source: PLoS One. 2025 Nov 25;20(11):e0337720. doi: 10.1371/journal.pone.0337720 (PMC12646400; doi:10.1371/journal.pone.0337720)

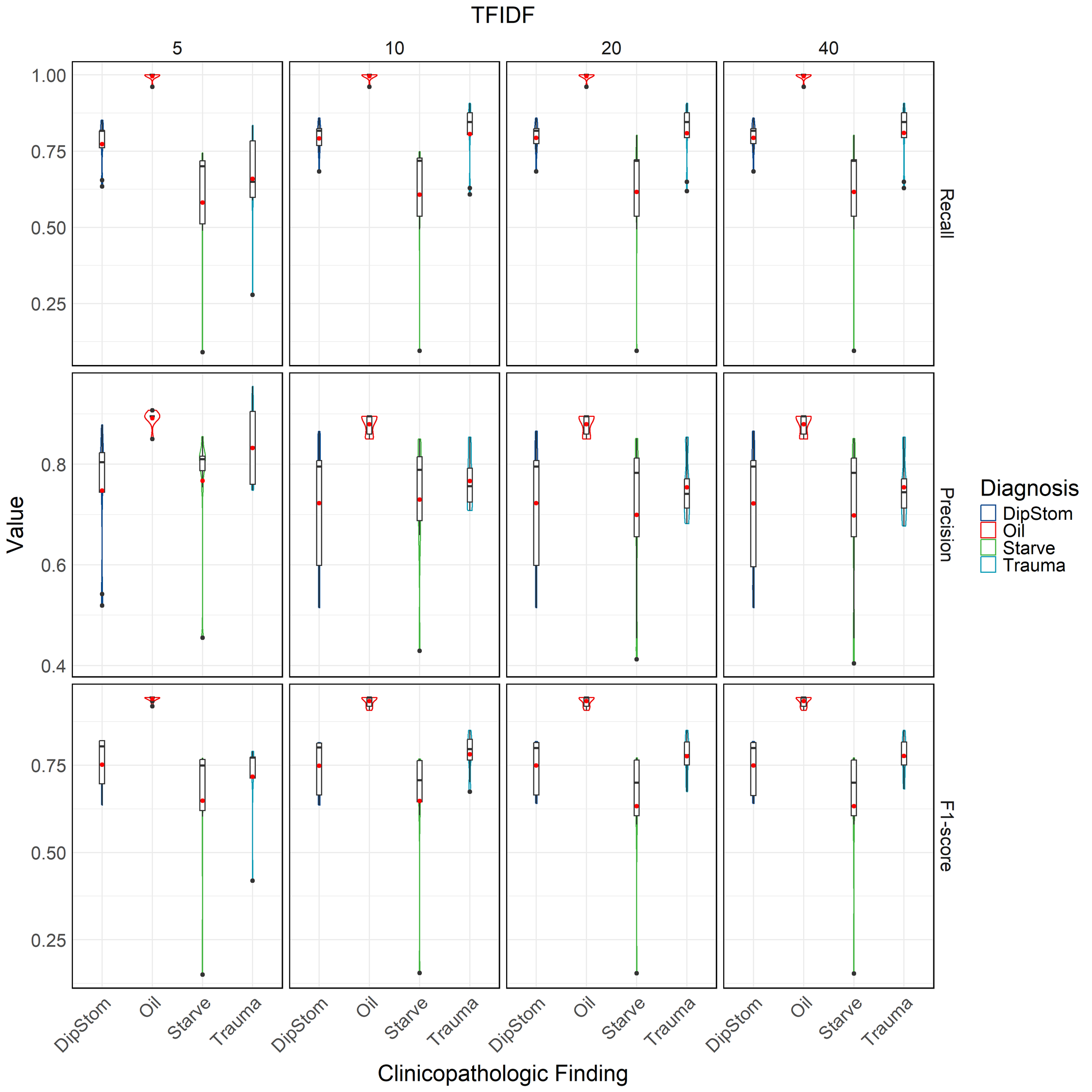

Supplement: S1 Fig — The distribution of performance metrics (right side labels and y-axis) across all testers (violin plots), clinicopathologic findings (x-axis), and each TFIDF condition (top labels) in a pilot test of the application DEE, designed for rapid extraction of necropsy data from the Wildbase Pathology Register of Aotearoa New Zealand. (TIF) [file pone.0337720.s003.tif]
